# Mechanisms of cancer cell killing by sea cucumber-derived compounds

**DOI:** 10.1007/s10637-017-0505-5

**Published:** 2017-09-18

**Authors:** Teresa Liliana Wargasetia, Widodo

**Affiliations:** 1 Faculty of Medicine, Universitas Kristen Maranatha (Maranatha Christian University), Jl. Prof. Drg. Suria Sumantri MPH No. 65, Bandung, 40164 Indonesia; 2 Biology Department, Faculty of Mathematics and Natural Sciences, The University of Brawijaya, Jl. Veteran, Malang, 65145 Indonesia

**Keywords:** Sea cucumbers, Triterpene glycosides, Anti-cancer, apoptosis, metastasis

## Abstract

The aim of cancer therapy is to specifically eradicate tumor cells while causing minimal damage to normal tissues and minimal side-effects. Because of this, the use of natural substances with low toxicity is a good option. Sea cucumbers are one of many potential marine animals that contain valuable nutrients and medicinal properties. The medicinal value of sea cucumbers is attributed to the presence of bioactive agents with promising biological and pharmacological properties that include cytotoxic activity, induction of apoptosis, cell cycle arrest, inhibition of tumor growth, anti-metastatic and anti-angiogenic properties, and inhibition of drug resistance. This review discusses the mechanisms of cancer cell death induced by sea cucumber-derived compounds with regard to exploring the potential use of these marine natural products for cancer therapy.

## Introduction

Cancer is the second leading cause of death worldwide, and the incidence is on the rise in both developing and developed countries [1]. The rapid development of resistance to cancer drugs, and the high toxicity and side-effects of some cancer chemotherapy drugs have caused an increase in the demand for new anti-cancer drugs, particularly from natural products [2]. For more than 40 years, natural products have played a prominent role in cancer treatment, either in their native or modified forms [3]. A 15-year survey conducted by the National Cancer Institute of the United States discovered that 4% of marine species tested (largely animals) contained anti-tumour properties [4]. Recently, the search for anticancer agents derived from marine natural products has increased [5].

Historically, the marine environment has proven a rich source of potent natural anti-cancer metabolites. Spongothymidine was the first marine-derived substance, found in 1945, to be developed for the treatment of acute myeloid leukemia and lymphoma. Currently, four other marine cytotoxic compounds, including eribulin, trabectedin, cytarabine, and vedotin, have been established as conventional clinical medicines [6].

Sea cucumbers (or holothurians), belonging to the class Holothuroidea, phylum Echinodermata, are marine invertebrates that are found in benthic areas and deep seas. They have a leathery skin and gelatinous body, shaped like soft-bodied cucumbers. Sea cucumbers, also called trepang, bêche-de-mer, gamat, balate, or haishen (marine ginseng), have long been utilized in food and Asiatic folk medicine [7, 8]. They contain valuable nutrients such as vitamin A, B1, B2, B3, magnesium, calcium, zinc, iron [8], triterpene glycosides (saponins, sti-choposides, frondoside A, cucumariosides, Ds-echinoside A) [8–14], fucoidan [15, 16], triterpenoid aglycones (philinopgenin) [17], non-glycosaminoglycan sulfated glycans [18], sulfated polysaccharides [19], non-sulphated triterpene glycosides (variegatusides) [20], sphingoid bases [21], and chondroitin sulfates [22].

Many researchers have reported anti-cancer activity of substances derived from sea cucumbers and have published the molecular mechanism of these agents in cancer cells. In this review, we will discuss the anti-cancer activities and molecular mechanisms of bioactive metabolites derived from sea cucumbers. This review is expected to provide new insight into the possible therapeutic activity of bioactive compounds derived from sea cucumbers for the treatment of many different types of cancers.

## Cytotoxicity activity

Anti-cancer agents exhibit cytotoxic activity through blocking or preventing growth or mitosis of cancer cells [5]. The cytotoxic activity of sea cucumber compounds in human tumor cell lines are well established. For example, holothurin A3 and A4, produced by the *Holothuria scabra* species, were discovered to be highly cytotoxic in human epidermoid carcinoma (KB) and human hepatocellular carcinoma (Hep-G2) cell lines [23]. Similarly, philinopside E, a newly discovered sulfated triterpene glycoside derived from *Pentacta quadrangularis*, showed potent cytotoxicity (IC_50_ = 0.75–3.50 μg/mL) in various tumour cell lines (human leukaemia cells-HL60, mouse lymphocytic leukaemia cells-P388, human non-small lung carcinoma cells-A549, gastric carcinoma cells-MKN28, lung adenocarcinoma cells-SPC-A4, gastric carcinoma cells-SGC7901, human epithelial carcinoma cells-A431, human hepatoma cells-BEL7402, human ovarian carcinoma-HO8901, and human fetal lung fibroblasts-W138) [24].

Sea cucumber compounds that induce cytotoxic effects in a broad range of cancer cell lines are presented in Table 1. Table 1 shows that triterpenoid glycosides (saponins) are the most abundant type of metabolites derived from sea cucumbers.

**Table 1:** Active compounds from sea cucumber

Compound	Species	Human cell line tested	Ref.
Aqueous extract	*Holothuria arenicola*	CT26	[25]
Arguside A	*Bohadschia argus*	HCT-116	[26]
Arguside B, C, D, E		A549, HCT-116, HepG2, MCF-7	[27] [28]
Bivittoside	*Holothuria polii*	HCT116, MCF7	[29]
Colochiroside A	*Colochirus anceps*	p388, HL60, A-549, SpC-A4, MKN28	[30]
Crude saponin	*Holothuria leucospilota*	B16F10	[31]
Cucumarioside A2–2	*Cucumaria japonica*	Ehrlich ascite carcinoma, HL-60	[32] [33]
Ds-echinoside A	*Peasonothuria graeffei*	Hep G2	[14] [34]
Echinoside A	*Holothuria nobilis*	26 human cell line	[35]
Echinoside A	*Peasonothuria graeffei*	Hep G2	[34]
Frondanol A5	*Cucumaria frondosa*	HCT116, AsPC-1, S2013	[36] [37]
Frondoside A and B		MDA-MB-23, A549,MDA-MB-435, MCF-7, HepG2, HL-60, AsPC-1, AsPC-1, S2013, AsPC-1	[38] [13] [39] [33] [40] [41]
Frondoside A	*Cucumaria okhotensis*	THP-1, HeLa, RT112, RT4, HT-1197	[42] [43]
Griseaside A	*Holothuria grisea*	HL-60, BEL-7402, Molt-4, A549	[44]
Hillasides A and B	*Holothuria hilla* Lesson	A549, MCF7, IA9, CAKI-1, PC-3, KB	[45]
Holothurin A3 and A4	*Holothuria scabra*	KB, Hep-G2	[23]
Okhotoside B1,B2,B3	*Cucumaria okhotensis*	HeLa cervical cancer	[42]
Organic/water extract	*Stichopus chloronotus*	C33A, A549	[46]
Pentactasides I,II, III, Philinopsides A and B	*Pentacta quadrangularis*	P-388, A-549, MCF-7, MKN-28, HCT-116, U87MG	[47]
Philinopsides E		P388, HL60, A549, SPC-A4, MKN28	[24]
Sphingoid bases	*Stichopus variegates*	Caco-2, DLD-1, WiDr	[21]
Sphingoid bases	Sea cucumber	HepG2	[48]
Stichoposide D	*Thelenota anax*	HL-60, K562	[49]
Violaceusides A and B	*Pseudocolochirus violaceus*	HL-60, BEL-7402	[50]
Stichorrenoside C and B	*Stichopus horrens*	Hep-G2, KB, LNCaP, MCF7, SK-Mel2	[51]

A431 human epithelial carcinoma cells, A549 non-small lung carcinoma, AsPC-1 pancreatic cancer, BEL-7402 hepatoma, B16F10 melanoma, Caco-2 colon cancer, CAKI-1 renal cancer. CT26 colon carcinoma, C33A cervical cancer, DLD-1 colon adenocarcinoma, HCT-8 colorectal adenocarcinoma, HCT116 colon cancer, HeLa cervical cancer, Hep G2 hepatocellular liver carcinoma, Hep3B hepatoma, HL-60 leukemia, HO8901 ovarian carcinoma, HT-1197 urothelial carcinoma, IA9 ovarian ca, KB epidermoid cancer, KB-VIN vincristine-resistant, K562 leukemia, LNCaP prostate cancer, LNM35 lung cancer, MCF7 breast adenocarcinoma, and MDA-MB-231 Luc-2 breast cancer, MDA-MB-435 breast cancer, MKN-28 gastric cancer, Molt-4 leukemia, NCI-H460-Luc2 lung cancer, PC-3 prostate cancer, p388 leukemia, RT4 urothelial carcinoma, RT112 urothelial carcinoma, TCCSUP urothelial carcinoma, THP-1 leukemia, T-24 urothelial carcinoma, SGC-7901 gastric cancer, SK-Mel2 melanoma, SPC-A4 lung adenocarcinoma, S2013 pancreatic cancer, U87MG glioblastoma, WiDr colon carcinoma, W138 human fetal lung fibroblast, 486p urothelial carcinoma

## Induction of apoptosis

Apoptosis is the process of programmed cell death that is characterized by cell shrinkage and morphological changes including membrane blebbing, chromosomal DNA fragmentation, nuclear disintegration, loss of organelle position in the cytoplasm, and translocation of phosphatidylserine to the outer membrane leaflet [52, 53]. Apoptosis results in the removal of unwanted, old, or injured cells including cancer cells and is, therefore, an important mechanism for tumor suppression [53, 54]. For this reason, reductions in apoptosis or apoptotic resistance play important roles in carcinogenesis. In general, the mechanisms by which reductions in apoptosis or resistance to apoptosis occurs are due to 1) disruptions in the balance between pro-apoptotic and anti-apoptotic proteins, 2) inactivation or loss of caspase activity, or 3) impaired death receptor signaling [55].

Induction of apoptosis is one the most prominent markers of cytotoxic antitumor agents. It has been shown that some natural compounds isolated from sea cucumbers induce apoptotic pathways through several different mechanisms to inhibit cancer progression. Marzouqi et al. (2011) found that frondoside A, a saponin isolated from *Cucumaria frondosa*, increased the sub-G1 (apoptotic) cell fraction through increases in p53, and subsequent induction of the caspase 9 and 3/7 cell death pathways in breast cancer cells [38]. Frondoside A also demonstrated strong anti-tumor activity through induction of apoptosis, increased expression of p21, and through increased activity of caspase 3, 7, and 9 in AsPC-1 and S2013 human pancreatic cancer cell lines [39]. Treatment with Frondanol A5, an isopropyl alcohol/water extract of *Cucumaria frondosa*, induced apoptosis that was associated with H2AX phosphorylation and led to cleavage of caspase-2 in HCT116 colon cancer cell lines. This study demonstrated the chemopreventive effects of Frondanol A5 against the development of colon cancer [36]. Another study found that a polar fraction of Frondanol A5 potently induced apoptosis in pancreatic cancer cells, AsPC and S2013 [37]. Philinopside A and E, glycosides isolated from *Pentacta quadrangularis* **,** reportedly induced apoptosis in mouse Sarcoma-180 tumors and tumor-associated endothelial cells [56, 57]. Stichoposide C, isolated from *Thelenota anax*, was found to induce apoptosis as demonstrated by mitochondrial injury and induction of signaling pathway disruption of cancer cells in human leukemia and mouse colorectal cancer cells. In this study, Stichoposide C induced apoptosis in a dose-dependent manner through activation of Fas, caspase-3 and caspase-8, cleavage of Bid, and mitochondrial damage in cancer cells [58].

Increased levels of reactive oxygen species (ROS) induce cells to undergo apoptosis. Administration of methanolic extracts of *Holothuria parva* led to increased ROS production, mitochondrial swelling, mitochondrial membrane potential (MMP), and cytochrome C release in the mitochondria in an animal model of hepatocellular carcinoma [59]. Induction of apoptosis and other mechanisms related to anticancer activity induced by metabolites isolated from sea cucumbers are summarized in Table 2.

**Table 2:** Relevant anti-cancer cell effects induced by sea cucumber compounds

Compound	Cell effect	Source	Ref.
24-dehydroechino-side A (DHEA)	Inhibit HepG2 cell migration and invasion, decrease MMP-9, increase TIMP-1, VEGF, & NF-κB (by HA_1_)	*Pearsonothuria graeffei*	[60]
Aqueous extract	Attenuate tumor size, induce intrinsic apoptosis	*Holothuria arenicola*	[25]
Colochiroside A	Exhibit antitumor activity	*Colochirus anceps*	[30]
Crude saponin	Induce apoptosis: upregulation of caspase-3 & 9	*Holothuria leucospilota*	[31]
Cucumarioside A2–2	Inhibit growth of tumor cells multidrug resistance, induce apoptosis in a caspase-dependent way	*Cucumaria japonica*	[32] [33] [61]
Ds-echinoside A	Suppress migration, MMP-9, and VEGF; cell cycle arrest (increase p16, p21, and c-myc; decreased cyclin D1), induce apoptosis by decreased Bcl-2, NF-κB, and increase TIMP-1 & caspase-3	*Peasonothuria graeffei*	[14] [34]
Echinoside A	Induce apoptosis (decrease Bcl-2, and enhance caspase-3), cell cycle arrest (increase p16, p21, and c-myc, decrease cyclin D1)	*Holothuria nobilis Peasonothuria graeffei*	[34] [35]
Frondanol A5	Increase p21, GiLT expression, macrophage phagocytosis & apoptosis	*Cucumaria frondosa*	[36] [62]
Polar extract of frondanol A5	Decrease expression of cyclin A, cyclin B, and cdc25c, and increased expression of p21	*Cucumaria frondosa*	[36]
Frondoside A	Anti-metastatic (by antagonize EP4, TPA-induced MMP-9 activation via NF-κB and AP-1 signaling), inhibit tumor cells multidrug resistance, induce apoptosis (via caspase-3, −8, and −9, PARP, Bax, p21, p53), inhibit pro-survival autophagy	*Cucumaria frondosa*	[13] [38] [39] [43] [40] [41] [61] [63] [64]
Glycosides 1 & 2	Activate NF-kappaB and degrade Ikappa B alpha in A549 tumor cell line (cytotoxic activity)	*Psolus patagonicus*	[65]
Intercedensides A, B, and C	Exhibit anti-neoplastic activity	*Mensamaria intercedens*	[66]
Methanolic extracts	Increase ROS formation, and cytochrome c release	*Holothuria parva*	[59]
Philinopside A	Inhibit proliferation, migration, angiogenesis, RTKs in several cell lines	*Pentacta quadrangulari*us	[56]
Philinopside E	Induce cell apoptosis, inhibit tumor growth, anti-angiogenic via inhibition of KDR-α*v*β3 integrin	*Pentacta quadrangulari*us	[57] [67]
Sea cucumber fraction	Exhibit radical scavenging property, inhibit angiogenesis, and vascularization	*Sea cucumber*	[68] [69]
Sphingoid bases	Induce apoptosis through caspase-3 activity, upregulation of DR5, Bax, GADD45, and PPARγ, and downregulation of p-AKT	*Stichopus variegates*	[21] [48]
Stichoposide C	Induce apoptosis of leukemia and colorectal cancer cells	*Thelenota anax*	[58]
Stichoposide D	Induce cell apoptosis	*Thelenota anax*	[49]

## Cell cycle arrest

Mammalian cells progress through several cell cycle phases (G1, S, G2, and metaphase) during cellular division. Cell cycle checkpoints, notable features of cell cycle progression, provide essential surveillance to prevent cells from entering the next phase before the previous phase has been completed [70]. Thus, halting the cell cycle can lead to prevention of cancer cell growth and division and is one of the major strategies for cancer therapy [71].

Cucumarioside A2–2 demonstrated anticancer effects through its ability to cause the arrest of the cell cycle during the DNA synthesis (S) phase and was shown to induce programmed death in Ehrlich carcinoma mouse tumor cells [32]. In another study, Echinoside A and Ds-echinoside A, triterpenoid glycosides isolated from *Peasonothuria graeffei*, caused the arrest of the cell cycle during the G0/G1 phases in hepatocellular liver carcinoma cells (HepG2). A reverse transcriptase-polymerase chain reaction assay showed that both triterpenoid glycosides increased expression of cell-cycle-related genes, including p16, p21, and c-myc, and decreased expression of cyclin D1 [34]. Frondanol A5, an isopropyl alcohol/water extract of *Cucumaria frondosa*, inhibited growth during the S and G2/M phases and led to increased levels of p21WAF1/CIP and decreased levels of Cdc25c [36]. A polar fraction of Frondanol A5 was effective at inducing G2/M cell cycle arrest, and also potently decreased expression of cyclin A, cyclin B, and Cdc25c in S2013 and AsPC-1 cells [37].

## Reduction of tumor growth

Tumour growth inhibition by Frondoside A was initially demonstrated in a xenograft model using AsPC-1 pancreatic cancer cells [38]. Frondoside A was also shown to inhibit tumour growth and reduce tumor volume by 87% in an athymic mouse model using MDA-MB-231 breast cancer cells [63].

Philinopsides A and E, novel sulfated triterpenoid glycosides derived from *Pentacta quadrangulari*, were also shown to reduced tumor growth. Philinopside A reduced tumor growth in the sarcoma 180 mouse model, whereas philinopside E inhibited tumor growth in both sarcoma 180 and hepatoma 22 mouse models [56, 57]. Other glycosides that demonstrate the ability to reduce tumor growth are summarized in Table 2.

## Anti-metastatic and anti-angiogenic effects

Metastasis and angiogenesis are the most dangerous processes acquired by cancer cells. Metastasis is the development of secondary malignant growths at a distance from the primary site of the tumour and one of the principal causes of mortality in cancer patients [14]. Angiogenesis is the formation of new blood vessels from pre-existing vessels that involves the growth, migration, and differentiation of endothelial cells, which line the inside walls of blood vessels. Angiogenesis occurs in tumors to help them to survive and proliferate. Anti-angiogenic agents inhibit this process, thereby preventing the supply of oxygen and nutrients from reaching cancer cell such that tumor cells starve and eventually die [72]. The ability of the cancer drugs to inhibit metastasis and angiogenesis is essential for effective cancer treatment strategies.

Ds-echinoside A, a compound isolated from *Pearsonothuria graeffei,* caused reductions in cell adhesion, migration, and invasion in HepG2 cells. Immunocytochemical studies revealed that treatment with Ds-echinoside A led to decreased expression of matrix metalloproteinase-9 (MMP-9) and resulted in increases in both angiogenesis and metastasis through degradation of the extracellular matrix. Ds-echinoside A also enhanced expression of tissue inhibitors of metalloproteinase-1 (TIMP-1), a crucial regulator of MMP-9 activation. The anti-angiogenic effect of Ds-echinoside A was also studied in a series of in vitro and in vivo tumor models. These studies showed that Ds-echinoside A decreased tube formation of human endothelial cells ECV-304 grown in Matrigel and attenuated neovascularization in a chick embryo chorioallantoic membrane (CAM) model [14].

Similar results were obtained by Zhao et al. (2010), in which two sulfated triterpene glycosides, Holothurin A (HA) and 24-dehydroechinoside A (DHEA), from *Pearsonothuria graeffei* were identified*.* Both of these glycosides showed anti-metastatic effects both in vitro and in vivo. The study revealed that HA and DHEA administration reduced adhesion of HepG2 cells and human endothelial cells (ECV-304) grown in Matrigel and inhibited HepG2 cell migration and invasion in a dose-dependent manner. Both compounds suppressed tube formation of ECV-304 cells in Matrigel and attenuated neovascularization using the CAM assay. Immunocytochemical analysis showed that both HA and DHEA significantly reduced expression of MMP-9 and increased expression of TIMP-1. Western blot analysis revealed that both compounds markedly suppressed the expression of vascular endothelial growth factor (VEGF). Inhibition of VEGF is a key indicator of anti-angiogenic behavior, displayed by HA and DHEA compounds. Furthermore, only HA downregulated expression of nuclear factor-kappa B (NF-κB). NF-κB regulates expression of MMP-9 such that inhibition of NF-κB activation suppresses MMP-9 and tumor invasion [73]. This finding suggests that the anti-metastatic activity of saponins derived from *P. graeffei* could be mediated through NF-κB-dependent or -independent pathways, depending on their chemical structures [60].

A study by Ma et al. found that Frondoside A had potent anti-metastatic activity in a syngeneic murine model of metastatic breast cancer. Intraperitoneal administration of Frondoside A in mice implanted with mammary tumors in mammary glands, inhibited tumor metastasis to the lungs. Along with the anti-metastatic activity described in vivo*,* Frondoside A also inhibited migration of tumor cells in vitro through inhibition of prostaglandin E receptors, EP2, and EP4 [63].

Other studies have also reported that Frondoside A has the promising anti-metastatic potential for breast cancer therapy. Park et al. investigated the anti-metastatic effects of Frondoside A in MBA-MB-231 human breast cancer cells, and found that Frondoside A inhibited TPA-induced activation of AP-1 and NF-κB and decreased TPA-induced activation of ERK1/2, PI3K/Akt, and p38 MAPK signals caused a reduction in expression of MMP-9 [64]. In addition, Frondoside A decreased invasion of MDA-MB-231 tumour cells in a Matrigel invasion assay [38].

Frondoside A was shown to inhibit angiogenesis as indicated by reduced CD31 staining, used to measure microvessel density, in LNM35 lung cancer xenografts and was shown to block basal and bFGF-induced angiogenesis using the CAM assay. Frondoside A treatment demonstrated significant inhibition of metastasis in a lung cancer xenograft model and an in vitro Matrigel invasion assay, without toxic side effects [13].

## Inhibition of drug resistance

Resistance to chemotherapy is a major problem for cancer therapeutics. One of the major factors that contribute to chemotherapy resistance is a process called autophagy. Pharmacological inhibitors of autophagy are important to prevent the development of resistance to anti-cancer drugs. Frondoside A, a triterpenoid glycoside from *Cucumaria okhotensis,* inhibited pro-survival autophagy in human urothelial carcinoma cell lines resistant to standard therapies [43].

Another study showed that Frondoside A isolated from the *Cucumaria okhotensis* and cucumarioside А2–2 isolated from *Cucumaria japonica* decreased multidrug resistance in the Ehrlich ascites carcinoma mouse tumor model. Frondoside A or cucumarioside А2–2 both demonstrated the ability to form a complex with cholesterol to block membrane transport P-glycoprotein activity in tumor ascites derived from the mouse Ehrlich carcinoma tumor model, resulting in decreased multidrug resistance [61].

## Conclusion

Bioactive compounds isolated from the sea cucumber for use as anti-cancer agents has attracted the attention of cancer researchers because of their natural origin and long history as a nutritious food. Sea cucumbers contain many marine-derived agents that have the potential to inhibit the growth of several different types of human tumor cells as demonstrated in in vitro studies, in vivo murine models, and human studies. Several secondary metabolites derived from sea cucumbers demonstrate anti-cancer properties through multiple mechanisms including cytotoxic activity, induction of apoptosis, cell cycle arrest, reduction of tumor growth, suppression of invasion and metastasis of tumor cells, inhibition of angiogenesis, and decreased drug resistance. Nevertheless, detailed mechanisms of the anti-cancer activities of sea cucumber derived-compounds remain unclear and comprehensive studies that identify these mechanisms are needed. Above all, the potential anti-cancer activity of bioactive compounds isolated from sea cucumbers gives promising hope for the treatment and prevention of human cancers.
